# Special Issue “The Role of Toll-Like Receptors (TLRs) in Infection and Inflammation 2.0”

**DOI:** 10.3390/ijms25179709

**Published:** 2024-09-07

**Authors:** Ralf Kircheis, Oliver Planz

**Affiliations:** 1 Syntacoll GmbH, 93342 Saal an der Donau, Germany; 2 Institute of Cell Biology and Immunology, Eberhard Karls University Tuebingen, 72076 Tuebingen, Germany; oliver.planz@uni-tuebingen.de

Toll-like receptors (TLRs) are key players in the innate immune system, in host’ first-line defense against pathogens [1,2]. TLRs recognize pathogen-associated molecular patterns (PAMPs) from bacteria, viruses, and other microorganisms, and self-derived damage-associated molecular patterns (DAMPs) released from dying or lytic cells [3,4,5,6,7,8,9]. as well as chromatin-associated molecular patterns (CAMPs) [10], thus playing a critical role not only in immune surveillance but also in disease pathogenesis [3,4,5,6,7,8,9,10,11,12]. The TLR activation triggers the production of proinflammatory cytokines and type I interferons, which are key mediators of the host immune response against bacterial, viral, and fungal infections [13,14,15,16,17,18]. However, dysregulation and excessive activation can be detrimental, leading to hyperinflammation, sepsis, and loss of tissue integrity. TLRs are involved in the pathogenesis of acute bacterial and viral infections, as well as chronic infectious and non-infectious inflammatory diseases [19,20,21,22,23,24].

This Special Issue 2.0 continues the conceptional line started in the previous Special Issue [25], focusing on the involvement of Toll-like receptors (TLRs) and their related signaling pathways in viral infection and inflammation, and emphasizing the therapeutic potential of TLR system modulation for the treatment of acute viral infections and their long-term sequelae and to provide potential targets for treating chronic viral and inflammatory diseases. This Special Issue provides an excellent collection of contemporary reviews and research articles covering a broad variety of topics, ranging from the protective activity of TLRs in viral infections to detrimental disturbances of the host immune system. The most intriguing theme discussed in this second Special Issue is the intercorrelation between acute viral infections and triggered delayed effects, including neuroinflammatory and neurodegenerative long-term sequelae.

The first review [26] provides an excellent overview on TLRs, triggered signaling cascades, and their function in immune surveillance and disease pathogenesis (see Figure 1). The authors discuss recent advancements in TLR research and the use of specific agonists and antagonists with application in immunotherapy and vaccine development. The authors illustrate the activation of specific TLRs, e.g., TLR7 and TLR9, in antiviral therapies or as adjuvants in cancer vaccines, as well as the targeting of TLR2 and TLR4 with antagonists to mitigate hyper-inflammatory responses in sepsis and viral infections, including COVID-19 and HSV-1 infection. Finally, the review describes the role of the RAGE/TLR system in diabetic encephalopathy, cancer, and cardiovascular diseases. Furthermore, the connection between innate and adaptive immunity and their impact on autoimmune diseases and neuroimmune diseases are discussed.

Finally, the work provides an outlook to future directions, such as the integration of computational modeling and personalized medicine approaches [26]. Computational modeling could represent a breakthrough in revolutionizing TLR research by providing tools for predicting TLR signaling pathways and analyzing the impact of potential therapeutics by simulating TLR modulation in silico before proceeding with in vivo studies [27,28,29,30,31,32].

The second review [33] provides a comprehensive overview on the Toll-like receptor response to Human Immunodeficiency Virus Type 1 (HIV-1) and co-infection with Hepatitis B or C Virus. HIV-1 is known to modulate the host TLR response with impact on viral persistence or disease resolution, e.g., HIV co-infection is associated with a higher persistence of HBV co-infection, higher HBV DNA levels, and increased cirrhosis and liver-related mortality, as well as a higher risk of hepatocellular carcinoma (HCC) [34,35,36]. Similarly, HIV co-infection can also aggravate the clinical course of HCV, resulting in higher HCV persistence, higher viral loads, enhanced liver fibrosis [37,38,39,40,41], and the development of HCC [42,43]. Increased expression of TLR6, TLR7, and TLR8 or TLR2, TLR3, and TLR4 mRNA has been observed in chronic untreated HIV-1 infections and in patients with advanced disease, respectively, demonstrating the involvement of TLRs and their signaling during HIV infection. TLR7 polymorphisms were shown to modulate the progression of HIV-1 infection. A decreased secretion of Th1 cytokines such as IL-2 and antiviral interferons, together with an increased secretion of Th2 cytokines, i.e., IL-4 and IL-10, and proinflammatory cytokines TNF-α, IL-1, IL-6, and IL-8 have been found in HIV-1 infection, correlating with disturbances in TLR activation. HIV regulatory proteins, HIV-1 Tat and Nef, have been demonstrated to modulate cytokine release and TLR activation, leading to enhanced viral replication [44,45]. Furthermore, HIV-1 accessory proteins Vif, Vpr, and Vpu were shown to inhibit cellular restriction factors and the TLR sensing of HIV-1 [46,47,48]. Activation of TLR signaling during HIV infection and the effect on viral replication are shown in Figure 2.

Finally, the potential of TLR agonists as latency-reverting agents and immune stimulators and their use in new strategies to cure HIV are discussed. Thus far, a persisting HIV-1 reservoir in long-lived memory CD4+ T cells, with a transcriptionally silent provirus that remains undetected by the host immune system, is seen as the highest challenge to a cure for HIV-1 [49]. Although ART can suppress HIV-1 replication to undetectable levels, it cannot eliminate latent persistent viral reservoirs. For a complete cure of HIV-1, new strategies must be developed that eliminate latently infected cells persisting in people with HIV on ART [50,51]. In this regard, latency-reversing agents that can induce viral reactivation, leading to immune cell recognition and the elimination of latently infected cells, will have a key role [52,53]. TLR agonists as key modulators of the host immune response could act as latency-reversing agents in HIV-1 and HCV/HBV co-infected patients [54].

The third review discusses the latest knowledge on the involvement of TLR in SARS-CoV-2 infection and COVID-19 [55]. TLR activation has been shown to contribute to viral clearance and disease resolution [56,57]. This is also illustrated by a three times higher probability of male patients for severe clinical course and death compared to females, and this may be related to the X chromosome-linked TLR7 gene with a higher and more stable TLR7-driven IFN-α production in female dendritic cells protecting from progression to severe disease [58,59,60].

However, TLRs represent a double-edged sword that can elicit dysregulated immune signaling exacerbating dysregulated immune response in patients with severe COVID-19. TLR2, TLR3, TLR4, and TLR7 have been associated with COVID-19 severity [61]. Different components of SARS-CoV-2 were shown to activate different TLRs (see Figure 3). The E protein was shown to activate TLR2/TLR1 and TLR2/TLR6, and the S protein has been demonstrated to activate TLR4 correlating with excessive NF-κB activation, cytokine release, and immune dysregulation [62,63,64,65]. Furthermore, SARS-CoV-2 was suggested to bind and activate TLR4, leading to increased ACE2 expression, correlating with enhanced viral entry and hyperinflammation [66]. TLR7/8 and TLR3 are activated by the viral ssRNA and the dsRNA intermediate, respectively. The TLR3 receptor has been widely described as a host defense factor against different viruses [67,68]. An in silico study has indicated the NSP10 protein of SARS-CoV-2 to activate TLR3 [69]. The authors discuss the role of TLRs in the pathogenesis of COVID-19 with the examples of TLR7 and TLR3 rare variants, such as the L412F polymorphism in TLR3 that negatively regulates anti-SARS-CoV-2 immune responses [70]. The authors also discuss the interaction of TLR2 and TLR4 with SARS-CoV-2 proteins and involvement of TLR2 in NET formation by SARS-CoV-2 [71].

Overall, in addition to its pivotal role, for an effective antiviral response, excessive TLR-triggered hyperinflammation can alter the equilibrium that drives disease severity [55].

Furthermore, Pedicillo et al. [72] demonstrate the association between TLR-4-triggered inflammation and macrophage imbalance in lung inflammatory infiltrates of lung tissue autopsies from lethal COVID-19 cases. The persistence of the spike protein was shown to correlate with TLR4 upregulation and increase macrophage infiltration. Importantly, a macrophage shift characterized by a downregulation of GAL-3(+) alveolar macrophages, which are crucial for resolving inflammation and promoting tissue repair, paralleled by an increase in CD163(+) myeloid-derived monocyte-macrophages, was shown. The data indicate that TLR-4 expression and activation induce persistently active inflammation, with inefficient resolution, and pathological macrophage shifts, as part of the pathophysiological mechanisms of lethal COVID-19 [72].

The critical role of TLR4 in COVID-19 pathophysiology is further supported by in silico studies analyzing the S protein-induced dimerization of TLR4/MD-2 complexes of the SARS-CoV-2 Omicron variant in comparison to wild-type virus and earlier SARS-CoV-2 variants [73]. This work addresses the question of why the pathogenicity of Omicron variants is significantly lower than that of wild-type virus and earlier Variants of Concern (VoCs). Despite a binding activity to TLR4 that is compatible to that of the wild-typer virus, a lower potency of the Omicron spike protein to trigger the dimerization of TLR4/MD-2 complexes was found. This can explain Omicron’s lower pathogenicity compared to wild-type virus and VOCs to a large extent. Furthermore, in silico data indicate the tendency of a decreasing TLR4 dimerization potency starting earlier, during SARS-CoV-2 evolution, somewhere after the transition from wild-type or Alpha variant to Gamma and Delta variants; however, this became most pronounced with the appearance of Omicron variants [73]. Figure 4 illustrates the consequences of TLR4 hyperactivation by the spike protein, particularly of wild-type SARS-CoV-2, compared to the lower activation of the Omicron variants.

Besides TLR4, another widely expressed and extensively studied pattern recognition receptor is TLR2. TLR2 is unique in that it forms heterodimers with TLR1, TLR4, TLR6, or TLR10, which allows it to recognize a very broad range of pathogens. The study by Colleselli et al. [75] in this Special Issue provides an overview of TLR2, its homo- and heterodimers, and of the pro- and anti-inflammatory properties of TLR2. The review provides an interesting outline of TLR2-associated infectious diseases, such as sepsis, COVID-19, and neurodegenerative diseases, such as Parkinson’s disease and Alzheimer’s disease (see Figure 5) [76,77,78,79,80,81].

The article by Abarca-Merlin et al. [82] provides an excellent overview on the involvement of TLRs in immunity and neurogenesis and on Toll-like receptors as regulators in the nervous system. TLRs are expressed in resident immune cells as well as in neurons and glial cells of the nervous system (see Figure 6). There is further support for the role of TLRs not only in the immune response but also in physiological and pathophysiological processes of the nervous system, such as neurogenesis, behavior, cognition, infection, neuroinflammation, and neurodegeneration [82,83,84].

The next work of Dubik et al. [85] on the involvement of TLR in neuroinflammation studied the synergistic targeting of TLR7 and NOD2 for therapeutic intervention in multiple sclerosis. Neuroinflammation regulation is critical for maintaining central nervous system (CNS) homeostasis and can be particularly relevant for the treatment of autoimmune diseases, including multiple sclerosis (MS). Earlier studies highlighted the significance of innate signaling in triggering anti-inflammatory mechanisms, playing a protective role in experimental autoimmune encephalomyelitis (EAE), which represents a predictive model for a MS-like disease. This study investigated the effects of targeting two innate receptors, TLR7 and NOD2, simultaneously to prevent EAE progression. The simultaneous intrathecal administration of NOD2 and TLR7 agonists led to the synergistic induction of Type I IFN and effectively suppressed EAE. The suppression of EAE correlated with a significant decrease in the infiltration of monocytes, granulocytes, and natural killer cells; reduced demyelination; and the downregulation of IL-1β, CCL2, and IFN-γ gene expression in the spinal cord. These results show the potential of targeting the innate TLR-related pathways for alleviating neuroinflammation associated with MS [85].

Hernandez et al. [86]’s article covers neurocognitive impairments in association with LPS-induced acute respiratory distress syndrome (ARDS) and the modulation of the Lung–Brain Axis of communication in wild-type vs. Fat-1 mice. Although ARDS primarily affects the lungs, many ARDS patients also develop neurocognitive impairments. To investigate the connection between the lung and brain during ARDS and the therapeutic potential of specialized pro-resolving mediators (SPMs), fat-1 mice were crossbred with Resolvin E1 (RvE1) receptor knockout mice. ARDS was induced through the intratracheal application of lipopolysaccharide. Protein and mRNA analyses revealed that LPS induced lung inflammation as well as increased inflammatory activation in the hypothalamus. The authors demonstrated that immune cell trafficking to the brain largely contributed to immune-to-brain communication during ARDS rather than cytokines. Deficiency in RvE1 receptors and enhanced omega-3 polyunsaturated fatty acid levels (fat-1 mice) were shown to affect lung–brain interaction during ARDS by altering the profiles of inflammatory and lipid mediators and glial activity markers [86].

The second group of articles focus on the role of TLRs in metabolic and cardiovascular diseases. Understanding the complex interactions between metabolism and the immune system may help us identify key immunomodulatory factors as therapeutic targets in obesity and cardiovascular diseases. The work of Höpfiner et al. [87] investigated the regulation of Cathelicidin Antimicrobial Peptide (CAMP) gene expression induced by TNFα and cfDNA in adipocytes. CAMP is a regulator of innate immunity expressed in adipocytes. CAMP, therefore, might act as an adipokine in adipose inflammation. TNFα, cell-free nucleic acids (cfDNAs), and TLR9 are all parts of the innate immune system and are functionally active in adipose tissue. Adipocyte inflammation was induced in vitro by TNFα and cfDNA stimulation. TNFα significantly induced CAMP gene expression in mature adipocytes, which was reduced through the inhibition of PI3K signaling. These findings suggest a regulatory role of inflammatory mediators, such as TNFα and cfDNA, in adipocytic CAMP expression as a novel putative molecular mechanism in adipose tissue innate immunity [87].

Mylonas et al. [88] studied the role of MYD88 and proinflammatory chemokines in aortic atheromatosis in the context of novel statin treatment. Their study investigated how statins mitigate proatherogenic inflammation. Statins, such as Rosuvastatin significantly reduced MYD88, CCL4, CCL20, CCR2, TNF-α, IFN-β, IL-1b, IL-2, IL-4, IL-8, and IL-10, both in the thoracic and abdominal aorta. Another statin, Fluvastatin, downregulated MYD88, CCR2, IFN-β, IFN-γ, IL-1b, IL-2, IL-4, and IL-10 in both aortic segments. In conclusion, statin therapy can control the proatherogenic inflammation in hyperlipidemic animals [88].

Finally, a very interesting work by Loaiza et al. studied the impact of extracellular histones and absence of Toll-like receptors on cardiac functional and electrical disturbances in mouse hearts [89]. Sepsis is frequently accompanied by cardiac functional and electrical disturbances. As a causative factor, extracellular histones, e.g., released from activated neutrophils, were shown to significantly contribute to cardiac dysfunction, as demonstrated by Echo-Doppler measurements. This study investigated the roles of extracellular histones and their interactions with TLRs in cardiac dysfunction through in vivo Echo-Doppler studies on mice perfused with extracellular histones in wild-type vs. TLR2, TLR3, or TLR4 knockout (KO) mice. Histone perfusion caused defects in contractility and relaxation, with TLR2 and TLR3 KO mice being partially protected. Specifically, TLR2 KO mice exhibited the greatest rescue effect. In contrast, TLR4 KO exacerbated cardiac dysfunction. Among individual histones, H1 induced the most pronounced abnormalities in cardiac function, cardiomyocyte apoptosis, and LDH release. The study indicates the interaction of histones with TLRs as potential therapeutic targets for septic cardiomyopathy [89].

Overall, this Special Issue 2.0 provides a diverse collection of original articles and review articles covering different aspects of Toll-like receptors and their roles in infection and inflammation. The various articles illustrate well the two sides of TLRs as drivers for the pathogenesis of acute bacterial and viral infections, including COVID-19; as drivers of multiple non-infectious diseases of various tissue origin; as potent players for antiviral, antibacterial immunity; and as therapeutic targets to treat chronical non-infectious inflammatory diseases [90,91,92,93,94,95,96]. Notably, new data increasingly show that neurological pathways, such as neurogenesis, neuroinflammation, and cognitive and behavioral aspects may be influenced by innate, TLR-mediated pathways. The SARS-CoV-2 envelope protein was shown to trigger depression-like behaviors and dyssomnia via TLR2-mediated neuroinflammation in mice [97], and the SARS-CoV-2 spike protein was shown to induce TLR4-mediated long-term cognitive dysfunction recapitulating post-COVID-19 syndrome in mice [98]. In this context, the connection between infection-triggered acute disbalance in TLR activation and resulting neurological sequelae following bacterial (sepsis) or viral infections, including long COVID-associated neurological sequelae [99,100,101,102], may provide new targets for future treatment options [103].

TLRs are drivers for both the immune response and the pathogenesis of acute bacterial and viral infections, but they are also involved in a broad variety of non-infectious diseases of neurological, metabolic, and cardio-vascular origin. The interconnection of infection-triggered TLR disbalances and resulting long-term neurological, cardio-vascular, and metabolic sequelae may represent promising targets for the development of new therapies.

## Figures and Tables

**Figure 1:**
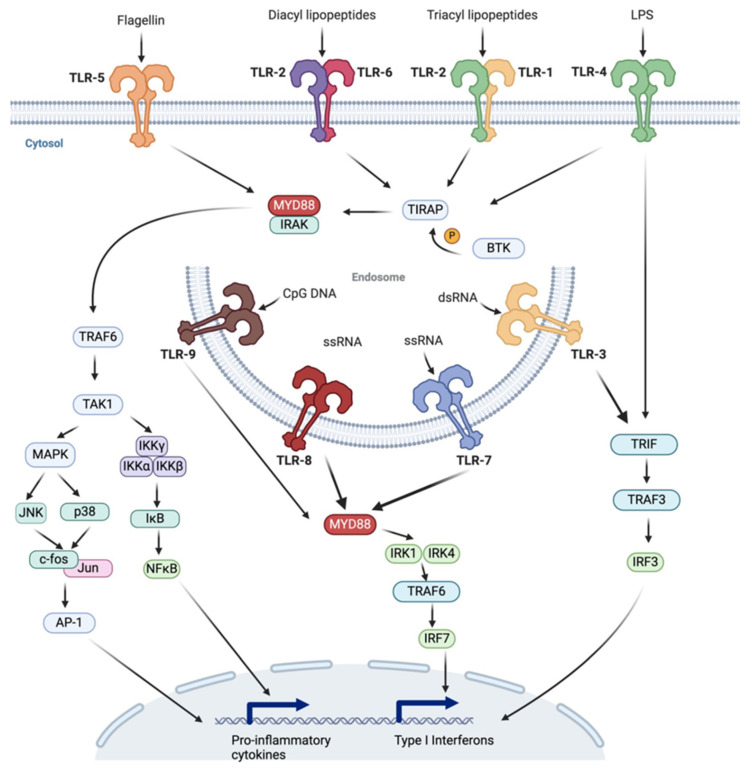
Overview of Toll-like receptor (TLR) signaling pathways showing cell surface TLRs (TLR-1, TLR-2, TLR-4, TLR-5, and TLR-6), their ligands (flagellin, diacyl lipopeptides, triacyl lipopeptides, LPS), and the endosomal TLRs (TLR-3, TLR-7, TLR-8, and TLR-9) recognizing CpG DNA and RNA molecules. The subsequent signaling cascades involving adaptor proteins such as MYD88, TRIF, and TRAF6 are shown below. These lead to the activation of NF-κB and IRFs and result in the expression of proinflammatory cytokines and type I interferons. Adopted from [26].

**Figure 2:**
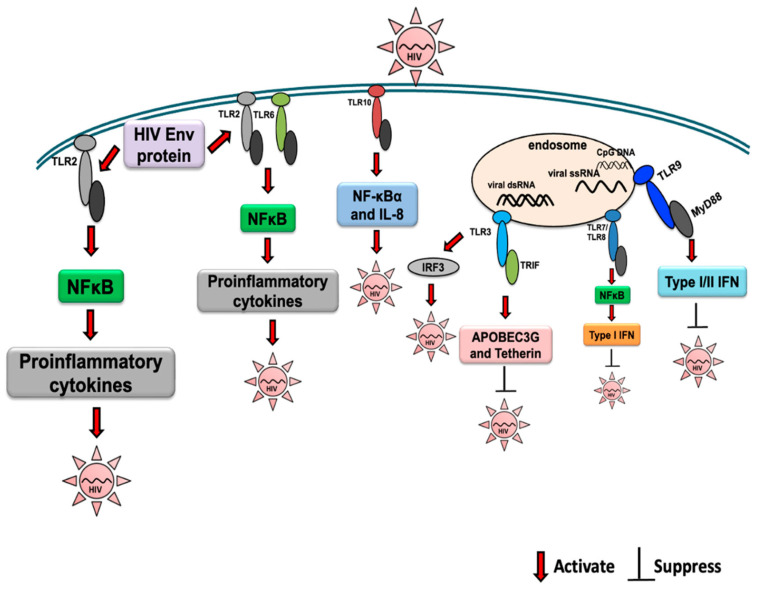
Activation of TLR signaling during HIV infection and the effect on viral replication are depicted. The activation and suppression of signaling are indicated with arrows. Adopted from [33].

**Figure 3:**
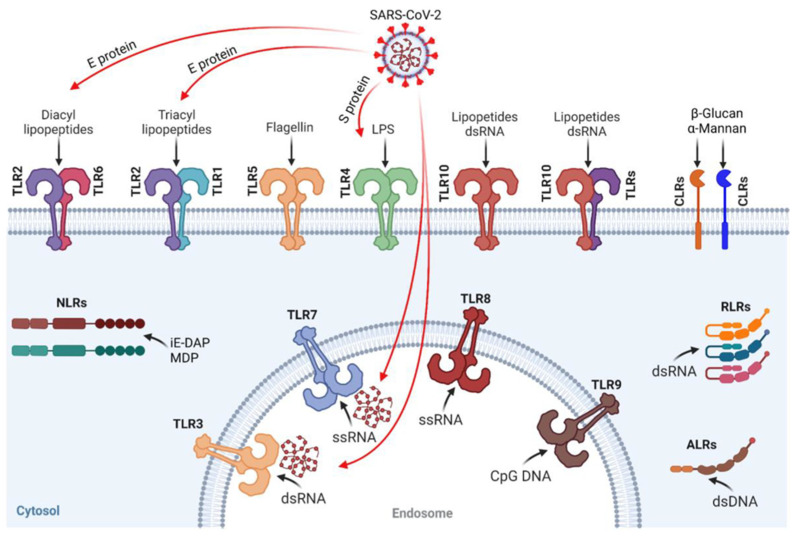
TLR activation by structural proteins of SARS-CoV-2. Adopted from [55].

**Figure 4:**
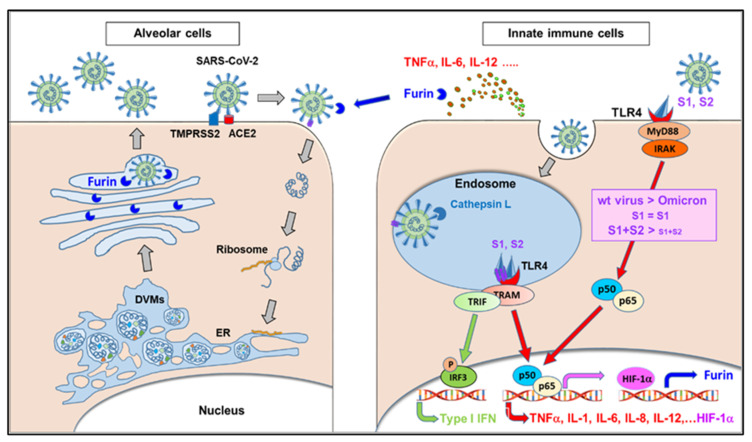
The SARS-CoV-2 binds to ACE2 followed by proteolytic cleavage by TMPRRS2 and fusion with the host cell membrane followed by the uptake of viral RNA into the host cell. The transcription of the viral RNA and translation of viral proteins occurs in double-membrane vesicles (DMVs). The viral components assemble into virus particles that leave the cells via the Golgi apparatus, where the spike protein undergoes proteolytic cleavage by furin. This process preferably occurs in virus-producer cells with high TMPRSS2 expression, e.g., alveolar cells (left side). Alternatively, the virus can be taken up via clathrin-coated pits into endosomes, followed by proteolytic cleavage by cathepsin L. The endosomal uptake is predominant in TMPRSS2-negative, cathepsin L-rich cells, such as innate immune cells (right side). The spike protein of the SARS-CoV-2 acts as a TLR4 agonist, resulting in the dimerization of the TLR4/MD-2 complex triggering downstream signaling, e.g., the NF-κB (p50/p65) pathway. The activation of the NF-κB pathway triggers HIF-1α activation and the expression of cytokines, such as TNFα, IL-1, IL-6, and IL-12. Notably, HIF-1α and IL-12 have been found to enhance furin expression. In contrast to the highly effective TLR4/MD-2 dimerization by the wild-type spike protein trimer, the amino acid substitutions in the Omicron spike protein interfere with the potency of the spike protein for TLR4/MD-2 dimerization, leading to less NF-κB signaling and lower cytokine expression. Adopted from [73,74].

**Figure 5:**
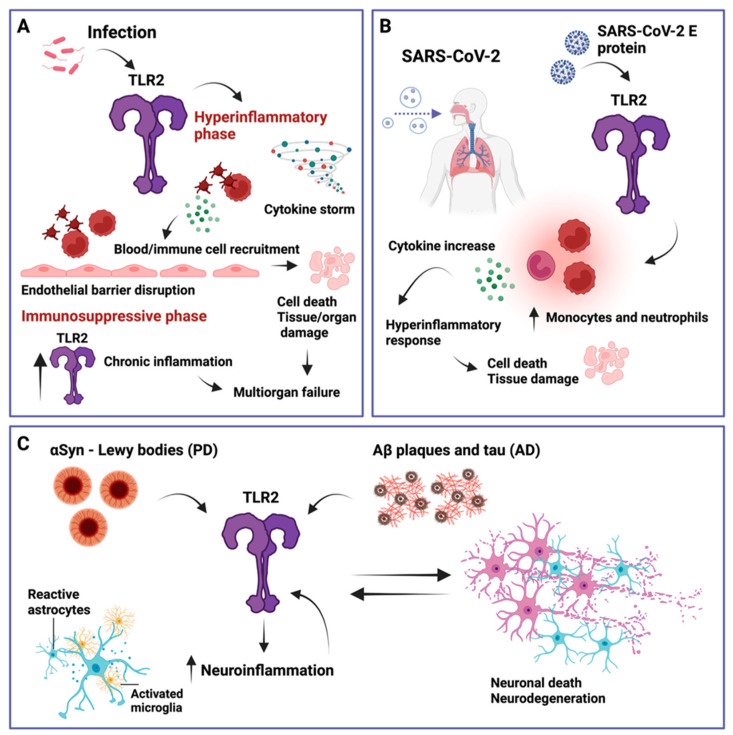
Overview of TLR2 involvement in different infectious and non-infectious inflammatory diseases. (**A**) Sepsis can be triggered by TLR2, which further activates platelets and other immune cells, leading to endothelial barrier disruption, cell death, and tissue and organ damage. In addition, TLR2 has been shown to be elevated during the immunosuppressive phase of sepsis, which can lead to multiorgan failure. (**B**) Severe acute respiratory syndrome coronavirus 2 (SARS-CoV-2). TLR2 can recognize the SARS-CoV-2 envelope (E) protein, leading to an increase in inflammatory monocytes and neutrophils, inducing a hyperinflammatory response that can cause cell death and tissue damage. (**C**) Parkinson’s disease (PD) and Alzheimer’s disease (AD). TLR2 can recognize α-synuclein (αSyn), amyloid-β (Aβ) plaques, and tau. This promotes neuroinflammation characterized by reactive astrocytes and activated microglia, which further increase TLR2 levels, leading to a feedback loop of neuronal cell death, further TLR2 upregulation, and neuroinflammation. Adopted from [75].

**Figure 6:**
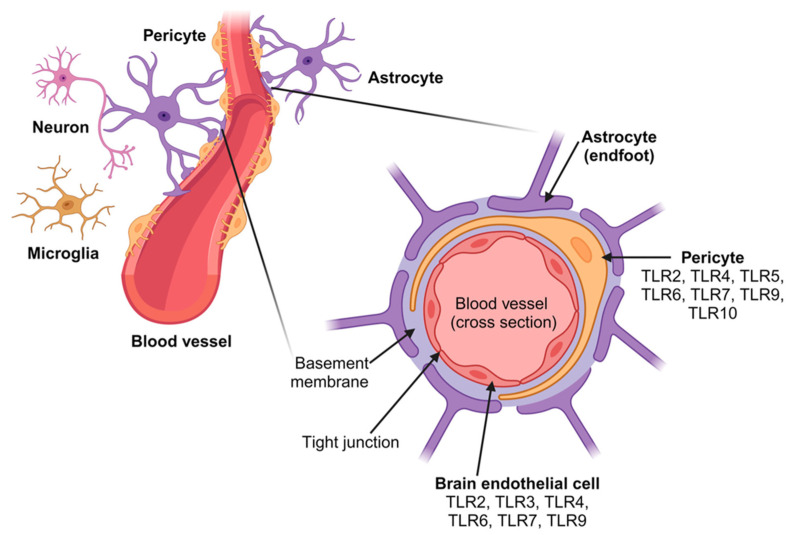
Schematic representation of the blood–brain barrier (BBB) located in the central part of the neurovascular unit (NVU). A cross-section of a blood vessel of the blood–brain barrier is depicted, showing the three main cell types that compose it (endothelial cells, pericytes, and astrocytes), as well as other cell types of the neurovascular unit (e.g., microglia and neurons) and their respective, expressed TLRs. The main function of the neurovascular unit is the formation of the blood–brain barrier and neurovascular coupling. Adopted from [82].
